# Effects of health poverty alleviation project from the perspective of vulnerability to poverty: evidence from five Chinese prefectures

**DOI:** 10.1080/16549716.2023.2260142

**Published:** 2023-10-02

**Authors:** Yan Wei, Zhaochi Zhang, Mingjian Zhang

**Affiliations:** School of Statistics, Xi’an University of Finance and Economics, Xi’an, China

**Keywords:** Health Poverty Alleviation Project, vulnerability to poverty, hierarchical linear regression, propensity score matching, China

## Abstract

**Background:**

The Health Poverty Alleviation Project (HPAP) has received widespread attention as a primary means of preventing poverty caused by illness. However, further evidence is required to confirm the effects of HPAP.

**Objective:**

This study examines the effectiveness and mechanisms of action of HPAP using data from a special survey conducted in five Chinese prefectures in 2018–2019.

**Method:**

This study uses a three-step feasible generalised least-squares method to measure the farm households’ vulnerability to poverty. Hierarchical linear regression and propensity score matching were employed to assess the poverty-reduction effects of HPAP. A mediating effects model was used to test how these policies alleviated poverty.

**Results:**

The mean vulnerability to poverty among farm households was 0.367, with 11.89% experiencing both poverty and vulnerability, particularly in areas of deep poverty. This study has found that HPAP significantly reduces poverty and is more effective in reducing the vulnerability of non-poor farm households than poor farm households. Additionally, the results suggest that improving human capital stock and reducing medical expenditure are the two pathways through which HPAP can alleviate farm households’ vulnerability to poverty.

**Conclusions:**

This study suggests that the vulnerability to poverty perspective should be incorporated into poverty alleviation policy formulation. HPAP enhances differentiation and precision. Thus, a long-term mechanism of HPAP should be developed.

## Introduction

The primary goal of the United Nations 2030 Agenda for Sustainable Development is to eradicate poverty [1], and China has achieved this goal 10 years ahead of schedule. Since 1978, China has reduced poverty by more than 70%, creating a Chinese model of poverty governance [2]. With the elimination of absolute poverty in China, the focus of poverty alleviation efforts has gradually shifted from economic to multidimensional poverty. Disease is a significant cause of poverty in China today, with approximately 40% of poverty arising from it [3]. Addressing health-related poverty is critical for eliminating poverty after achieving the goal of eliminating absolute poverty. China’s efforts in this area can provide valuable insights and experiences for developing countries seeking to reduce poverty further.

Poverty is defined as a state in which income or consumption falls below a certain level [4]. The limitation of this approach is that it only considers the current state of individual or household welfare at a given point and at a certain time but not future welfare and associated household risks. To overcome this shortcoming, the World Bank defined vulnerability to poverty as the probability of being poor or poorer in the future [5]. In contrast to traditional poverty measures, vulnerability to poverty can be used to describe the future status of poverty [6]. In academia, the three methods used for measuring include Vulnerability as Expected Poverty (VEP) [7], Vulnerability as Expected Utility (VEU) [8], and Vulnerability as Uninsured Exposure to Risk (VER) [9]. Currently, the method universally adopted by researchers is VEP, which measures the likelihood of a target individual or household falling into poverty in the future [7–10].

Health shocks have become the most important cause of vulnerability to poverty [11]. The essence of poverty is the deprivation of viability and loss of opportunity [12]. The lack of access to transportation, medical facilities, medical personnel, and health awareness in rural areas places rural residents at a high prevalence of health poverty [13]. In impoverished rural areas, where the healthcare system is often inadequate, the implementation of new rural cooperative medical system has often resulted in low reimbursement rates, limited coverage, and complicated reimbursement procedures. These factors contribute to catastrophic health expenditures for rural residents [14]. Health shocks, such as illness or injury, can not only increase medical costs and impose financial strain on households but also diminish the assets and future earning potential of individuals within the household. This diminishment is due to reduced labour time and efficiency caused by health shocks [15]. When a severe illness occurs within a household, it not only affects the productivity of the sick member but also reduces the economic income of the household by requiring other household members to take time off work or reduce their productivity to provide care for the sick member. This interaction between health and poverty creates a vicious cycle of ‘poverty caused by illness’ and ‘illness caused by poverty’ [16].

To end the cycle of illness and poverty, China has implemented the Health Poverty Alleviation Project (HPAP) since 2016. HPAP includes a series of policies aimed at implementing medical assistance and improving medical conditions [17]. The policies contained the guarantee of essential medical and health services for people in poverty, the improvement of public health, the improvement of medical institution services, and the optimisation of medical resource allocation [18]. By the end of 2020, nearly 10 million families that had fallen back into poverty due to illness had been effectively raised out of poverty [19]. Simultaneously, scholars have conducted extensive studies on HPAP. Some researchers have focused on the effects of the medical insurance system [20–22], whereas others have concentrated on the effects of the HPAP. Existing studies on the effects of the HPAP have primarily focused on what had been done rather than the effect on poverty alleviation [23,24]. Lu et al. [25] empirically analysed the economic effects of the HPAP using evidence from Chifeng City, China. However, whether HPAP exerts the same effect in other regions remains unknown.

In addition to China, numerous other governments worldwide have their own HPAP. Researchers have studied the relationship between these policies and individuals’ susceptibility to poverty. For example, Atake’s study in sub-Saharan Africa found that reducing healthcare costs or increasing medical insurance coverage could effectively improve vulnerability to poverty [26], and Vo and Van’s study in Vietnam found that health insurance could reduce vulnerability to poverty by 19% [27].

Despite the abundance of research on this topic, several gaps remain in our understanding of HPAP and its impact on farmers’ vulnerability to poverty. Specifically, there is a lack of research evaluating HPAP performance and the mechanisms through which these policies contribute to poverty reduction. Additionally, previous studies have often neglected the role of village-level or community variables in vulnerability to poverty. This study aims to address these shortcomings using special survey data to explore the relationship between HPAP and vulnerability to poverty and the pathways through which these policies impact poverty reduction.

## Materials and methods

### Variable selection and description

The dependent variable in this study is vulnerability to poverty, which is measured using the three-stage feasible generalised least squares (FGLS) method by calculating the log of income and the variance of the log of income. This measure represents the probability that a farm household will fall into poverty in the future and ranges from 0 to 1. The independent variable is whether a household is eligible for HPAP, which is indicated by a binary variable with a value of 1 for eligible households and 0 for ineligible households. The control variables include the characteristics of the household head (age, marital status, education level, and health status), characteristics of the household (population size, prevalence of severe or chronic illness, medical expenditure, presence of out-of-home workers, household labour force participation rate, and per capita arable land area), and characteristics of the village (per capita income, distance to the rural clinic, and ecological fragility). Ecological fragility refers to whether the village is located within an ecologically fragile area. The government designates the ecologically fragile areas accounting for more than 55% of China’s land [28]. The ecologically fragile areas are chartered with low stability, weak resistance, and high vulnerability [29] and often are related to poverty [30].

The characteristics of the variables used in this study are summarised in Table 1. In the sample, 43% of the farm households had eligibility for HPAP. The average age of the farm household head was 47.31, indicating a trend towards younger people leaving rural areas and older people remaining behind. Of the household heads, 84% had a spouse, and the average education level was above primary school, although the overall education levels were low. On average, the head of the household was in good health. The average size of farm households was 4.04 people, and the logarithm of per capita income was 8.53. The average prevalence of severe or chronic illness in farm households was 13%, indicating that the health of rural residents is not optimal. Medical expenses represented 11% of the total household expenditure, indicating a significant economic burden due to illness. At least one member was working out-of-home for 48% of farm households, and the household labour force participation rate was 61%, with an average per capita land area of 1.25 mu.^1^^1^1 mu = 666.67 m^2^ At the village level, the average coverage distance to the clinic was 2–4 km, and 27% of the farm households were located in ecologically fragile areas.

**Table 1.** Variables.

Category	Variable	Variable code	Definition	Mean	SD
Dependent variable	Vulnerability to poverty	vp	Measured results	0.37	0.03
Independent variable	Eligibility for HPAP	hpap	Yes = 1, No = 0	0.43	0.50
Characteristics of the household head	Household income per capita	Income_h	The logarithm of household net income (RMB)/the number of family members	8.53	1.08
	Age	age	Age of the head of the household	47.31	11.81
	Has spouse	spouse	Yes = 1, No = 0	0.84	0.37
	Education level	edu	No education = 1, Primary school = 2, Junior high school = 3, Senior high school/Secondary vocational School and above = 4	2.38	0.90
	Health status	health	Good = 1, Average = 2, Poor = 3	1.66	0.75
Characteristics of the household	Household size	size	The number of family members	4.04	1.40
	The prevalence of severe illness or chronic illness	twp	The total number of individuals who have severe illness or chronic illness/The number of family members	0.13	0.23
	The proportion of medical expenditure	med_exp	Medical expenditure/Household expenditure	0.11	0.17
	The presence of out-of-home workers	worker_ooh	Yes = 1, No = 0	0.48	0.50
	The proportion of the household workforce	wf_rate	The proportion of aged 15–59	0.61	0.22
	The arable land area per capita	land	Household arable land area (mu)/The number of family members	1.25	1.51
Characteristics of the village	Per capita income	income_v	The per capita income of the village	8.76	0.91
	Coverage distance of the clinic	clinic	Within 2 km = 1, 2–4 km = 2, Over 4 km = 3	2.16	0.79
	Ecological fragility	eco	Yes = 1, No = 0	0.27	0.44

### Study area and data resource

**Figure 1.**
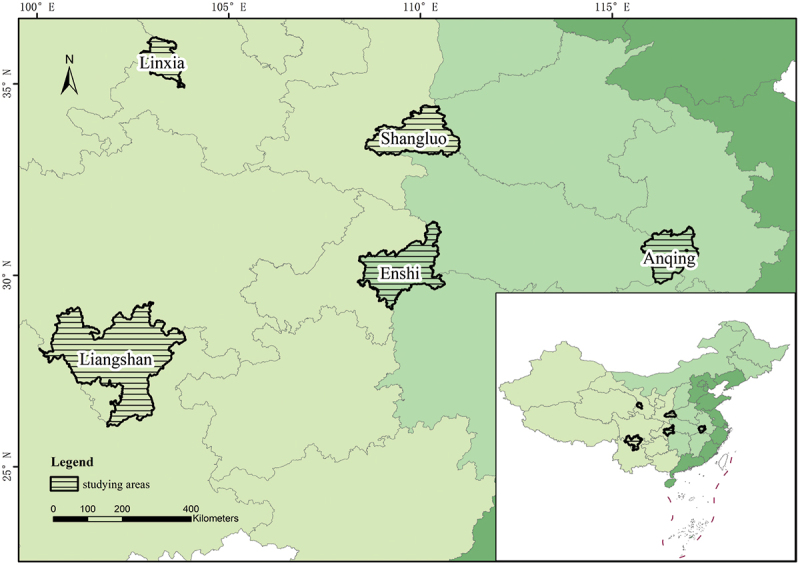
Locations of study areas.

The data for this study were collected during a special survey on ‘precise HPAP and population development’ founded by the National Social Science Foundation of China from December 2018 to February 2019. The survey targeted areas of deep poverty and key HPAP regions designated by the government. The study sites were selected from Liangshan Prefecture in Sichuan Province, Linxia Prefecture in Gansu Province, Anqing City in Anhui Province, Enshi Prefecture in Hubei Province, and Shangluo City in Shaanxi Province (Figure 1). These five sites were selected to represent HPAP implementation in China. Hubei and Anhui provinces are known for their large populations of people who have not yet escaped poverty caused by illness before 2018 [3]. Liangshan Prefecture in Sichuan Province and Linxia Prefecture in Gansu Province represent ‘The Most Impoverished Three Regions and Three Prefectures’. Shangluo City in Shaanxi Province was selected as a representative of a general poverty-stricken province for comparative purposes.

The survey was conducted by recruiting local college students with rural household registrations as investigators, who were trained and supervised by the subject team or coordinating teachers. Investigators returned to their hometowns to conduct the survey during the winter vacation, using a stratified sampling approach based on the registration forms of voluntarily enrolled students to cover as many regions as possible. A total of 252 college students from rural areas participated in the survey. The subject team followed up with some respondents and reviewed the returned questionnaires to ensure data quality. The survey included questionnaires on both household and village. The questionnaire on household covered five areas: basic family information and social support, family member information, health status and social support, satisfaction with medical services, and perception of HPAP. The questionnaire on village covered four areas: basic personal information of village officials, basic village information, living conditions, regional environment, and rural clinic hygiene. The survey first randomly extracted poor households defined by national rural poverty alleviation standards in each province and then extracted a random sample of non-poor households from the same villages. The sample size of poor households is the same as that of non-poor households. The final valid sample size was 2102. Based on the missing data, a final sample of 1295 farm households and 148 villages were analysed. A comparison of the survey data with data from the Sixth National Health Service Survey revealed good consistency (Table 2). The Sixth National Health Service Survey is conducted by the National Health Commission and covers all 31 provinces on the mainland. It is China’s largest family health survey, so the consistency indicates that the survey could represent HPAP implementation in China.

**Table 2.** Survey data of five Chinese prefectures compared with that of the sixth National health Service survey (%).

	Survey			Sixth National Health Service Survey		
	Average	Middle China Anqing, Enshi	West China Liangshan, Shangluo, Linxia	Average	Middle China	West China
Two-week prevalence	30.4	34.3	27.1	32.2	31.8	32.3
Two-week consultation rate	22.3	25.2	19.9	24.0	21.4	25.2
Health check attendance in the past year	18.1	20.2	16.3	47.2	44.9	47.7
Basic medical insurance coverage proportion	87.2	84.8	89.1	96.8	96.4	97.6
BMI						
Underweight (BMI < 18.5)	6.7	6.1	7.2	7.9	7.7	9.3
Normal (BMI = 18.5–23.9)	62.5	61.7	63.2	54.8	54.8	56.1
Overweight (BMI = 24–27.9)	26.2	28.2	24.6	28.9	29.2	26.9
Obese (BMI ≥ 28)	4.6	4.0	5.0	8.4	8.3	7.7

### Measurement of vulnerability to poverty

This study employed the VEP method, which was further refined by Bronfman et al., to measure vulnerability to poverty among farm households [31]. To control heteroscedasticity, the three-stage FGLS method was used to estimate the mean and variance of the logarithm of farm household income. First, the income function was estimated through the regression of the log of household income per capita (Equation (1)), and thereafter, the squared residuals from this regression were used to approximate income fluctuations in the subsequent regression (Equation (2)). The heteroscedasticity matrix was constructed as weights, and the weighted regression of the log of income and squared residuals was re-estimated to obtain the FGLS estimates of the mean ($X_{i}{\hat{\beta }}_{FGLS}$) and variance ($X_{i}{\hat{\theta }}_{FGLS}$) of the log of farm household income. According to the VEP theory, if we assume that farm household income follows a lognormal distribution, the farm households’ vulnerability to poverty can be calculated by setting the poverty line (Equation (3)).

(1) $$\begin{array}{c} Lnincome\_h_{i}=X_{i}\beta +e_{i} \end{array}$$

(2) $$\begin{array}{c} e_{i}^{2}=X_{i}\theta +\lambda_{i} \end{array}$$

(3) $$vp_{i,t}=\hat{\text{P}}(Lnincome\_h_{i,t+1}\leq \text{Ln}Z\text{|}X_{i}=\Phi \left(\frac{LnZ-X_{i}{\hat{\beta }}_{FGLS}}{\sqrt{X_{i}{\hat{\theta }}_{FGLS}}}\right)$$

In the above equations, $income\_h_{i}$ represents the income level of farm household i, $X_{i}$ represents a set of household- and village-level variables that influence the per capita income of the farm household, LnZ is the logarithm of the poverty line, and $vp_{i,t}$ represents the vulnerability to poverty of household i in period t. The selection of poverty and vulnerability lines plays a crucial role in the accuracy of vulnerability to poverty measurement. In this study, we used the 2018 national standard rural poverty line of RMB 3535 per person per year; and based on previous research, we set the vulnerability line at 0.5 [7]. This vulnerability line was also used in this study, with farm households whose probability of future poverty exceeds 0.5 as vulnerable and those below 0.5 as non-vulnerable.

### Hierarchical linear regression

To assess the impact of the HPAP on vulnerability to poverty and to consider the influence of both individual- and village-level covariates on the farm households’ vulnerability to poverty, this study used a hierarchical linear regression model implemented using HLM 8 software. Given that the future welfare status of farm households is affected by household- and village-level factors, each farm household can be nested within its corresponding village. The hierarchical linear model used to analyse the factors influencing the farm households’ vulnerability to poverty is as follows:

(4) $$\begin{array}{rl} & vp=\beta_{0}+\beta_{1}*hpap+\beta_{2}*age+\beta_{3}*spouse+\beta_{4}*edu\\ & \;\;\;\;\;\;\;\;\;+\beta_{5}*health+\beta_{6}*size+\beta_{7}*twp+\beta_{8}*med_{exp}\\ & \;\;\;\;\;\;\;\;\;\;+\beta_{9}*worker_{ooh}+\beta_{10}*wf_{rate}+\beta_{11}*land+\sigma^{2} \end{array}$$

(5) $$\begin{array}{c} \beta_{0}=\gamma_{00}+\gamma_{01}*income_{v}+\gamma_{02}*clinic+\gamma_{03}*eco \end{array}$$

(6) $$\begin{array}{c} \beta_{n}=\gamma_{n0}\left(n=1,2,\ldots ,12\right) \end{array}$$

Equation (4) examines farm household factors’ influence on vulnerability to poverty. Equation (5) examines the impact of village-level factors on farm households’ vulnerability to poverty by considering farm household-level factors. When considering the variables in Equation (5), the impact of these variables on the intercept in Equation (4) is actually examined.

### Propensity score matching

From the perspective of poverty dynamics, this study examined the effect of a farm household’s enjoyment of HPAP on its vulnerability to poverty to assess the impact of HPAP on poverty reduction. Since it is impossible to observe the state of enjoying HPAP and not enjoying it simultaneously, this study used a counterfactual causality approach for the analysis. The main idea of the Propensity Score Matching (PSM) method is to identify a control group through feature matching and thereafter make the experimental and control groups highly similar in characteristics through PSM. Thus, the difference in vulnerability to poverty after matching can be attributed to the impact of the HPAP, and the balancing and common support hypotheses are satisfied before matching. Logit regression models were used with Stata to estimate the probability of eligibility for HPAP, and the Average Treatment Effect (ATT) was calculated.

(7) $$\begin{array}{c} e\left(X_{i}\right)=P\left(hpap_{i}=1|X_{i}\right)=E\left[hpap_{i}|h\left(X_{i}\right)\right] \end{array}$$

(8) $$\begin{array}{rl} & ATT=E\left[vp_{1i}-vp_{0i}|hpap_{i}=1\right]\\ & \;\;\;\;\;\;\;\;\;\;\;=E\left\{E\left[vp_{1i}-vp_{0i}|hpap_{i}=1,e\left(X_{i}\right)\right]\right\}\\ & \;\;\;\;\;\;\;\;\;\;\;=E\{E[vp_{1i}\left|\begin{array}{c} hpap_{i}=1,e\left(X_{i}\right)]-E\\ \left[vp_{0i}|hpap_{i}=1,e\left(X_{i}\right)\right] \end{array}\right|hpap_{i}=1\} \end{array}$$

In Equation (7), the conditional probability of enjoying HPAP was denoted, where $policy_{i}$ denotes whether the household enjoys HPAP. ATT is calculated using Equation (8), where $vp_{1i}$ denotes the vulnerability to poverty of the household with HPAP and $vp_{0i}$ stood for those without it.

### Mediating effect model

This study used a mediating effect model to empirically test the mediating effects and mechanisms of the increment in human capital and out-of-pocket medical costs on the relationship between HPAP and vulnerability to poverty among farm households, based on the theoretical mechanisms of ‘HPAP improving human capital and reducing vulnerability to poverty’ and ‘HPAP reducing out-of-pocket medical costs and reducing vulnerability to poverty’. The mediating effect of the increment in human capital is measured using responses to the question ‘Health poverty reduction improves the quality of family labour’ in the household questionnaire. The proportion of out-of-pocket medical expenses among farm households is calculated as the proportion of out-of-pocket medical expenses to total household expenses.

(9) $$\begin{array}{c} vp=\lambda_{1}+\beta_{1}*hpap+\varepsilon_{1} \end{array}$$

(10) $$mv_{i}=\lambda_{2}+\beta_{2}*hpap+\varepsilon_{2}$$

(11) $$vp=\lambda_{3}+\beta_{3}*hpap+\beta_{3}*mv_{i}+\varepsilon_{3}$$

The above three equations were used to test these mediating effects and mechanisms, where $mv_{i}$ denotes the mediating variables (human capital growth and the out-of-pocket medical ratio).

## Result

### Measurement of vulnerability to poverty

The results for vulnerability to poverty and the distribution of the five municipalities are presented in Table 3. The mean vulnerability to poverty in the five regions is 0.367, which implies that the probability of farmers falling into poverty in the future is 0.367; 20.15% of farmers have a vulnerability over 0.5 and face a high risk of falling into poverty. Regarding regional distribution, the mean value of vulnerability to poverty and vulnerability proportions of Liangshan and Linxia were more significant than those of the other three regions. Moreover, the lower level of economic development, backward infrastructure construction, and high natural risks in Liangshan and Linxia indicate characteristics of greater vulnerability to poverty in deep poverty areas. Among the non-deep poverty areas, the mean vulnerability to poverty in Enshi was slightly higher than that in Anqing, Anhui. However, the vulnerability proportion in Anqing is higher, indicating that there are more highly vulnerable farmers in Anqing and significant regional differences in farm households’ vulnerability to poverty.

**Table 3.** Vulnerability to poverty by region.

Area	Vulnerability to poverty	Vulnerability proportion(%)
Linxia (*n* = 177)	0.435	31.1
Enshi (*n* = 306)	0.320	11.8
Anqing (*n* = 272)	0.310	12.5
Shangluo (*n* = 358)	0.371	19.3
Liangshan (*n* = 182)	0.460	37.9
Total (*n* = 1295)	0.367	2.2

### Baseline regression

Table 4 presents the results of the hierarchical linear regression of vulnerability to poverty for subsamples of poor and non-poor farming households. According to Table 4, the coefficients of the HPAP are all significantly negative, indicating that HPAP can significantly reduce farm households’ vulnerability to poverty and has a more significant effect on non-poor farm households. Among the control variables, the age of the household head positively affects vulnerability to poverty among farming households. However, marital status and education level were both significantly and negatively related to vulnerability to poverty. Farm households with married heads and higher education levels were less likely to experience poverty. Additionally, the poorer health status of a household was linked to a higher probability of poverty. The poorer the health status of the household head, the more likely the household is to fall into poverty. Moreover, owing to the poor health status and physical quality of the workers, they cannot adapt to high-intensity industrial development, which is one of the critical factors leading to long-term poverty and vulnerability of poor farm households; medical expenditures and the prevalence of severe or chronic illness can significantly increase the farm households’ vulnerability to poverty, and their regression coefficients are more prominent, indicating a more significant impact. The economic burden of disease and health shocks not only reduces households’ human capital stock but also leads to excessive catastrophic health expenditures. Farming households with larger populations are more likely to fall into poverty, whereas those with a higher labour force share are less vulnerable. This phenomenon is more pronounced among poor households; out-of-home workers alleviate non-poor households’ vulnerability but do not affect poor households. There is a positive relationship between village per-capita income and vulnerability to poverty, suggesting that the neighbourhood context influences vulnerability to poverty. Additionally, the proximity of rural clinics to farmers appears to have a protective effect on vulnerability to poverty, possibly due to the increased accessibility and availability of health services. Furthermore, farmers in ecologically fragile areas tend to experience higher levels of poverty and vulnerability, highlighting the importance of public health environment in impoverished areas for poverty-reduction efforts.

**Table 4.** Hierarchical linear regression estimation results.

Variables	Average	Poor	Non-poor
Fixed effects			
Intercept	0.723	0.735	0.716
Household variables			
Eligibility for HPAP	−0.018***	−0.016***	−0.020***
Age	0.001***	0.001***	0.001***
Has spouse	−0.113***	−0.115***	−0.111***
Education level	−0.069	−0.069***	−0.069***
Health status	0.001	0.003*	0.000
Population size	0.074***	0.077***	0.072***
The prevalence of severe illness or chronic illness	0.112***	0.115***	0.111***
The proportion of medical expenditure	0.319***	0.327***	0.300***
Presence of out-of-home workers	−0.001	−0.001	−0.002*
The proportion of the household workforce	−0.067***	−0.075***	−0.062***
Per capita arable land area	0.004***	0.005***	0.003***
Village-level variables			
Per capita income	−0.062***	−0.064***	−0.060***
Coverage distance of the clinic	0.033***	0.033***	0.032***
Ecological fragility	0.091***	0.094***	0.087***
Random effects			
Random term	0.007	0.007	0.006
Intercept reliability estimation	0.017	0.016	0.017

*p < 0.1, **p < 0.05, ***p < 0.01.

### The effect of HPAP

Before applying propensity score matching to obtain the ATT, it is necessary to conduct common support and balancing assumption tests. A common support test was performed on the sample to ensure that most of the treatment groups had matches in the control group. The kernel density curves of the treatment and control groups almost overlapped, satisfying the common support assumption of PSM. In other words, that means there are pairs of samples with almost characteristics of covariates, and the HPAP is the critical difference between the pairs. The balancing assumption requires that all covariates are balanced between the treatment and control groups with no significant differences, under the assumption of conditional exogeneity. The p-values of the post-matching t-tests were all greater than 0.1, and the deviations of all covariates after matching were less than 10%, satisfying the balancing assumption. The result of the balancing test means the pairs of samples mainly cover the treatment and control groups.

To eliminate the problem of self-selection bias, PSM was used to test the causal relationship between HPAP and vulnerability to poverty. In addition, the results of the vulnerability to poverty measure to determine whether the farmers are poor may have an impact on the above results, a sub-sample test was conducted using whether the farmers are poor as the basis for sub-sample division. Table 5 presents the average treatment effects of HPAP on vulnerability to poverty estimated using nearest neighbour matching, caliper matching, and kernel matching methods for the entire sample, non-poor farm households, and poor farm households. All three matching methods revealed a significant negative effect of the HPAP on the average treatment effect of vulnerability to poverty for different subgroups of farmers, indicating that HPAP can significantly reduce farmers’ vulnerability to poverty. Specifically, the ATTs of HPAP on the farm households’ vulnerability to poverty under the poverty-line criterion of RMB 3535 per year were −0.018, −0.030, and −0.019. For the subsamples of non-poor and poor farm households, overall, the effect of HPAP on the reduction of vulnerability of non-poor households was greater than that of poor households, and the effect of HPAP under caliper matching and kernel matching. There was a significant effect on the effect of vulnerability reduction for non-poor farmers with an ATT of −0.042 and −0.020, whereas there was no significant effect on the effect of vulnerability reduction for poor farmers. The impact of HPAP on poor farmers is limited because poor farmers find it more challenging to escape poverty and have a higher likelihood of falling vulnerable to poverty.

**Table 5.** The effect of HPAP.

	Analysis method	Treated	Control	ATT	T-value
Global	K-nearest neighbour matching	0.359	0.367	−0.018***	−1.76
	Caliper matching	0.350	0.380	−0.030***	−3.43
	Kernel matching	0.349	0.368	−0.019***	−2.03
Poor	K-nearest neighbour matching	0.432	0.441	−0.009	−0.48
	Caliper matching	0.432	0.434	−0.002	−0.10
	Kernel matching	0.432	0.452	−0.020	−1.17
Non-poor	K-nearest neighbour matching	0.308	0.320	−0.012	−1.07
	Caliper matching	0.308	0.350	−0.042***	−3.92
	Kernel matching	0.308	0.328	−0.020***	−1.98

*p < 0.1, **p < 0.05, ***p < 0.01.

### Mechanism of HPAP

Exploring the internal logic between HPAP and vulnerability to poverty is helpful for better understanding the relationship between the two, i.e. the ways in which HPAP reduces vulnerability to poverty. Based on the availability of data, a mediating effect test model of HPAP on farm households’ vulnerability to poverty was constructed using human capital increment and out-of-pocket medical ratio as mediating variables to further test the mechanism of the effect of HPAP on poverty reduction. In accordance with the steps of the mediating effect test model introduced in the previous section, Table 6 provides two groups comprising six models (Group 1 comprises Models 1–3 and Group 2 comprises Models 4–6) for testing the transmission mechanism of the poverty-reduction effect of health alleviation.

**Table 6.** Mediating effect model estimation results.

Group 1: Human capital growth	Model 1	Model 2	Model 3
HPAP	−0.019*** (0.001)	0.390*** (0.064)	−0.018*** (0.001)
Human capital growth	\	\	−0.001* (0.001)
Control variable	Control	Control	Control
Group 2: Out-of-pocket medical ratio	Model 4	Model 5	Model 6
HPAP	−0.023*** (0.005)	−0.021*** (0.006)	−0.021*** (0.006)
Out-of-pocket medical ratio	\	\	0.101*** (0.101)
Control variable	Control	Control	Control

*p < 0.1, **p < 0.05, ***p < 0.01; standard errors are in parentheses.

For the human capital increment effect in Group 1, Models 1 and 3 are tobit regressions with vulnerability to poverty as the explanatory variable and Model 2 is a multiple linear regression with human capital increment as the explanatory variable. The control variables are related variables such as the marital status of the household head, education level of the household head, land area per capita, and work outside the household. The results reveal that HPAP is negatively correlated with vulnerability to poverty among farming households in Model 1. In Model 2, HPAP is found to have a significant positive effect on human capital growth. In Model 3, HPAP and human capital increment negatively impact vulnerability to poverty among farming households. These findings suggest that efforts to address health poverty can reduce vulnerability to poverty among farming households and that increasing human capital through HPAP is also effective in reducing vulnerability to poverty. In summary, HPAP reduces farm households’ vulnerability to poverty by increasing their human capital.

To assess the effect of the out-of-pocket medical ratio in Group 2, Model 4 and Model 6 are tobit regression with vulnerability to poverty as the explanatory variable, and Model 5 is a multiple linear regression with the out-of-pocket medical ratio as the explanatory variable. The control variables were related variables, such as the health status of the household head, prevalence of severe or chronic illness, and the farthest distance covered by rural clinics. The results reveal that HPAP is negatively correlated with vulnerability to poverty among farmers in Model 4. In Model 5, HPAP is found to have a significant positive effect on the proportion of out-of-pocket medical expenses incurred by farmers. In Model 6, HPAP and the out-of-pocket ratio of medical expenses were found to significantly impact vulnerability to poverty among farmers. In summary, the mechanism by which the HPAP can alleviate vulnerability to poverty in farm households by reducing medical expenses was verified.

## Discussion

This study first measured vulnerability to poverty; thereafter, it evaluated the factors influencing vulnerability to poverty and the poverty-reduction effect of HPAP and finally explored the mechanism of the health poverty-reduction effect. The results reveal that HPAP significantly reduces households’ vulnerability to poverty in two ways: by improving human capital stock and by reducing medical expenditures.

The survey results demonstrate that some non-poor farm households also belong to the vulnerable group. Of the farm households, 8.26% were in a non-poor status, however, in a poverty-vulnerable state. The results of this study suggest that it is vital to consider both poor and non-poor farming households that are vulnerable, for poverty-reduction efforts. Of the poor farm households, 23.09% may come out of poverty stably, indicating that the poverty alleviation policy has achieved specific results; 11.89% of the farm households are not only poor at present but also face poverty in the future, and it is more difficult for these farmers to escape poverty, indicating that high vulnerability is accompanied by poverty.

Consistent with previous studies [32–34], this study indicates that households are significantly impacted by village-level variables. The boost in the per capita income of villages and rural clinics can reduce vulnerability to poverty, whereas a fragile ecological system increases it. The results reveal that when selecting aid targets, not only family characteristics but also village characteristics and environmental variables should be considered. Moreover, families with the same characteristics have different vulnerabilities to poverty due to different village-level characteristics. Future policies should focus on concentrated, contiguous, poverty-stricken, medically lacking, and ecologically fragile areas.

Based on the results of this study, initiatives focused on improving health and reducing poverty have a more notable effect on reducing vulnerability to poverty among households that are not currently impoverished as opposed to those that are already poor. This conclusion differs from previous research, suggesting that medical aid can significantly decrease vulnerability to poverty among low-income households [27]. This may be because there are many reasons for the poverty of poor farm households, and it is difficult to reduce their vulnerability only using HPAP effectively. It is necessary to formulate multidimensional assistance policies that can alleviate VEP from an overall poverty perspective [35]. HPAP is a long-term investment process. It is difficult to improve the health of the family members of poor farm households and quickly increase the stock of human capital. In addition, there may be dislocations and deviations in implementing HPAP, and effective assistance measures for farm households in poverty are required [36].

This study had certain limitations. First, this study should have considered differences in the prevalence of chronic and acute diseases. Some studies have revealed that HPAP may have different effects on VEP in patients with chronic or severe diseases [21]. Second, the limited duration of cross-sectional data used in this study may have negatively affected our conclusions. If possible, we will use additional panel data to extend our research in the future.

## Conclusions

This study used special survey data from five provinces in China to quantitatively examine the poverty-reduction effect of HPAP in poverty-stricken areas from the perspective of vulnerability to poverty and reached the following conclusions:

First, from the perspective of vulnerability to poverty, the stability of poverty alleviation in rural areas remains less optimistic, and there are noticeable regional differences. Deeply impoverished areas are primarily located along borders and in ethnic minority areas. The main characteristics include ecological fragility, frequent natural disasters, insufficient medical resources, and poor infrastructure. Farm households are more vulnerable to risks and have insufficient ability to resist shocks, which could easily lead to them becoming trapped in the ‘poverty trap’ cycle [16].

Second, HPAP has a significant poverty-reduction effect, which can effectively reduce the risk of farm households falling into poverty in the future and significantly reduce the vulnerability of non-poor farm households.

Third, increasing the stock of household human capital and reducing medical expenditures are two ways to effectively alleviate farm households’ vulnerability to poverty. Policies aimed at alleviating poverty in rural areas can have several positive impacts, including improving the overall health of residents, strengthening the public health system, increasing household labour productivity, and reducing farm households’ vulnerability to poverty [10]. Additionally, such policies can improve the medical security levels of farm households and provide transfer payment assistance, such as medical assistance, which can directly reduce catastrophic health expenditures and indirectly increase the income of other productive capital within households; it accumulates and reduces vulnerability to poverty [26].

## Paper context

The study started with the implementation of the Health Poverty Alleviation Project. The authors confirmed that the Health Poverty Alleviation Project could reduce the vulnerability to poverty especially in the non-poor, by improving human capital stock and reducing medical expenditure. It is suggested that the Health Poverty Alleviation Project should enhance differentiation and precision, and more measures should be introduced to prevent poverty.
